# Selection of reliable reference genes for qPCR studies on chondroprotective action

**DOI:** 10.1186/1471-2199-8-13

**Published:** 2007-02-26

**Authors:** Stefan Toegel, Wenwen Huang, Claudia Piana, Frank M Unger, Michael Wirth, Mary B Goldring, Franz Gabor, Helmut Viernstein

**Affiliations:** 1 Department of Pharmaceutical Technology and Biopharmaceutics, University of Vienna, Althanstrasse 14, A-1090 Vienna, Austria; 2 Beth Israel Deaconess Medical Center, New England Baptist Bone and Joint Institute, and Harvard Medical School, Boston, Massachusetts 02115, USA

## Abstract

**Background:**

Chondroprotective agents (CPA) such as glucosamine, curcumin and diacerein represent potential remedies for the management of osteoarthritis and several studies have been performed on their effects in-vitro and in-vivo. For the investigation of chondroprotective action on chondrocyte gene expression, quantitative real-time RT-PCR is the method of choice. However, validation of applied normalization strategies represents a crucial and sometimes neglected step in the analysis process. Therefore, the present study aimed to determine the expression stability of common reference genes (*ACTB*, Beta actin; *GAPDH*, Glyceraldehyde-3-phosphate; *B2M*, Beta-2-microglobulin; *HPRT1*, Hypoxanthine phosphoribosyl-transferase I; *SDHA*, Succinate dehydrogenase complex, subunit A; *YWHAZ*, Tyrosine 3-monooxygenase/tryptophan 5-monooxygenase activation protein, zeta polypeptide) under the influence of glucosamine, curcumin and diacerein in the IL-1β-stimulated C-28/I2 chondrocyte model, using the geNorm software tool.

**Results:**

CPA treatment of C-28/I2 chondrocytes significantly affected the expression level of many reference genes (p < 0.05). According to their expression stability, geNorm analysis revealed rankings of the 3 most stable genes (from most stable to least stable) as follows: *GAPDH, B2M *and *SDHA *in glucosamine treated samples and *HPRT1, GAPDH *and *B2M *in curcumin or diacerein treated samples. Interestingly, *ACTB *was one of the most variably expressed genes throughout all experiments.

**Conclusion:**

Our study points out the problem of relying on commonly used reference genes without an accurate validation process. For normalization purposes in gene profiling studies on glucosamine action, the genes *GAPDH, B2M *and *SDHA *are recommended as single reference genes depending on the expression level of the target gene or more favourably in combination. For experiments with curcumin and diacerein the use of *HPRT1, GAPDH *and *B2M *should be considered.

## Background

Osteoarthritis (OA) is a chronic, degenerative disorder of unknown cause characterized by gradual loss of articular cartilage. It is the most common of all joint diseases and represents a major social and economic burden since its prevalence increases with age [1]. Currently, classic therapeutic approaches are still limited to symptom relieving drugs or surgical intervention. Chondroprotection with drugs possessing disease-modifying qualities represents an alternative concept in the treatment of OA and its clinical potential has been the subject of numerous studies, both clinical and in-vitro [2-10]. Examples of chondroprotective agents (CPA) are glucosamine, chondroitin sulphate, curcumin, diacerein, rhein, and avocado/soybean unsaponifiables. However, medical opinion about the applicability and clinical efficacy of CPA in OA remains divided. In the case of glucosamine, for example, numerous clinical reports have indicated beneficial effects such as symptom relief in OA [2,3]. On the other hand, several reviews have provided criticism of study designs or processing [11,12]. Moreover, the recently published 'Glucosamine/chondroitin Arthritis Intervention Trial (GAIT)', which was intended to clarify the effectiveness of glucosamine and chondroitin in reducing the pain of osteoarthritis, has generated further controversy by revealing negative findings and high placebo response [13]. It should be further noted that to date limited information regarding synergistic effects, contraindications and drug interactions of CPA is available [14]. However, the rationale to intervene therapeutically in the course of OA with pharmacological agents able to improve biosynthetic processes of chondrocytes accounts for an ongoing interest in the evaluation of CPA. Moreover, these often natural-sourced agents are widely available, generally well tolerated and presumably possess more desirable safety profiles as compared to non-steroidal anti-inflammatory drugs (NSAIDS). A detailed clarification of the molecular effects of CPA is therefore regarded as a reasonable and promising approach leading to progress in understanding of OA modifying action.

At the molecular level, osteoarthritic tissue destruction originates from an imbalance of catabolic cytokine and anabolic growth factor pathways in chondrocytes and synovial cells. Studies have shown that the sensitive homeostasis of cartilage extracellular matrix is effectively disturbed by the TNFα/IL-1β driven cytokine cascade [15-17]. Recent in-vitro studies suggest that CPA have direct influence on the cytokine cascade resulting in down-regulation of catabolic downstream mediators such as collagenase, aggrecanase, NO synthase and prostaglandin E2 [8-10]. For the investigation of chondroprotective action, IL-1β-stimulated chondrocyte models represent a helpful tool [7,8,10,18-20]. In these models, chondrocytes cultured in monolayer or alginate beads are incubated with an inflammatory mediator, such as IL-1β and the CPA of interest, alone and in combination, and selected targets are investigated at the protein or gene expression level.

During the analysis of the molecular biological consequences of chondroprotective action, real-time quantitative RT-PCR (qPCR) is the method of choice for monitoring alterations of gene expression patterns in chondrocytes [21-25]. qPCR is one of the most powerful analytical tools for the sensitive and accurate quantification of mRNA expression levels in cells of different populations or under different culture conditions. One of the main challenges encountered in qPCR data interpretation is the selection of an appropriate normalization strategy. Data normalization is required to control for the experimental error introduced during the multistage process of isolating and processing RNA [26]. Different options range from standardization of tissue weight or cell numbers to the use of artificial molecules incorporated into the samples [26]. However, the most popular method for internal control of qPCR data error is the use of reference genes with presumably stable expression levels under a variety of experimental conditions. Since several studies have demonstrated the potential regulation of those reference genes by the conditions of bioassay [27-31], leading qPCR investigators have declared that the validation of reference genes for each experimental situation is a crucial requirement for the acquisition of biologically meaningful data [26,29,32,33]. In this context, a recent study demonstrated that when a carefully validated reference gene is used to normalize qPCR data, the results can be significantly different from those obtained when an unvalidated reference gene is used [34]. Nevertheless, a survey of the recent literature makes it apparent that reference genes are often used for normalization of qPCR data without any mention of a validation process. This is also true for papers on chondrocyte function. Furthermore, only two studies investigating reference gene stabilities in chondrocytes under mechanical loading or between patient samples, respectively, have been published [35,36].

The present study was designed to obtain a methodological evaluation of the influence of chondroprotective agents on reference gene mRNA levels in the IL-1β stimulated chondrocyte model. We therefore cultured IL-1β-treated C-28/I2 chondrocytes in the presence of selected CPA, i.e. glucosamine, curcumin, or diacerein and assessed the mRNA levels of prominent reference genes by qPCR. Our results will provide information for future studies on CPA about appropriate reference genes for internal standardization of qPCR data in the IL-1β stimulated chondrocyte model.

## Results

### Optimization and quality assessment of the qPCR protocol

Since the efficiency of reverse transcription reactions depends on the total amount of RNA, the input RNA for the reverse transcription step should be identical if various groups of samples are compared [26]. This requires accurate quantification of the isolated RNA as well as assessment of the RNA integrity. In the present study, RiboGreen assays of RNA isolated from differently treated C-28/I2 chondrocytes revealed RNA amounts ranging from 6.96 μg to 36.3 μg. No correlation between the amount of total RNA and the different treatments was found. The integrity of the RNA preparations was verified by the inspection of the 28S and 18S ribosomal RNA bands using agarose gel electrophoresis. Efficiency of the DNase digest was additionally assessed by implementing minus RT controls for each RNA preparation in the qPCR experiments. Some of the minus RT control samples recorded C_t _values of around 32 indicating minor genomic DNA contamination. However, the ΔC_t _– separating the minus RT controls from the unknown samples – of more than 13 C_t _was regarded as high enough to be negligible [37]. In summary, all RNA samples were found suitable for qPCR reactions.

Optimization of primer concentrations is critical for increasing specificity and sensitivity of the qPCR assay [33]. The results of the described primer optimization experiments are presented in Table 1. For all qPCR reactions of the study, these respective primer concentrations were used. Afterwards, standard curves were prepared to examine the quality of the overall qPCR assay. Standard curves of all examined genes revealed high amplification efficiencies over 4 to 5 orders of magnitude (Table 1) covering the C_t _range of all samples. The R^2 ^values of the standard curves exceeded 0.996. Gene-specific amplification was confirmed by a single peak in the melting-curve analyses (data not shown). Only *ACTB *amplification resulted in the detection of a second peak, which is known to originate from the presence of regions with highly different GC content rather than from unspecific reactions [38]. Moreover, no primer-dimer was detectable in any of the melting-curves indicating optimal performance of the primers. The no-template controls (NTC), included in all qPCR runs, did not record any positive C_t _value throughout the study.

### Transcriptional profiling of the reference genes

**Figure 1:**
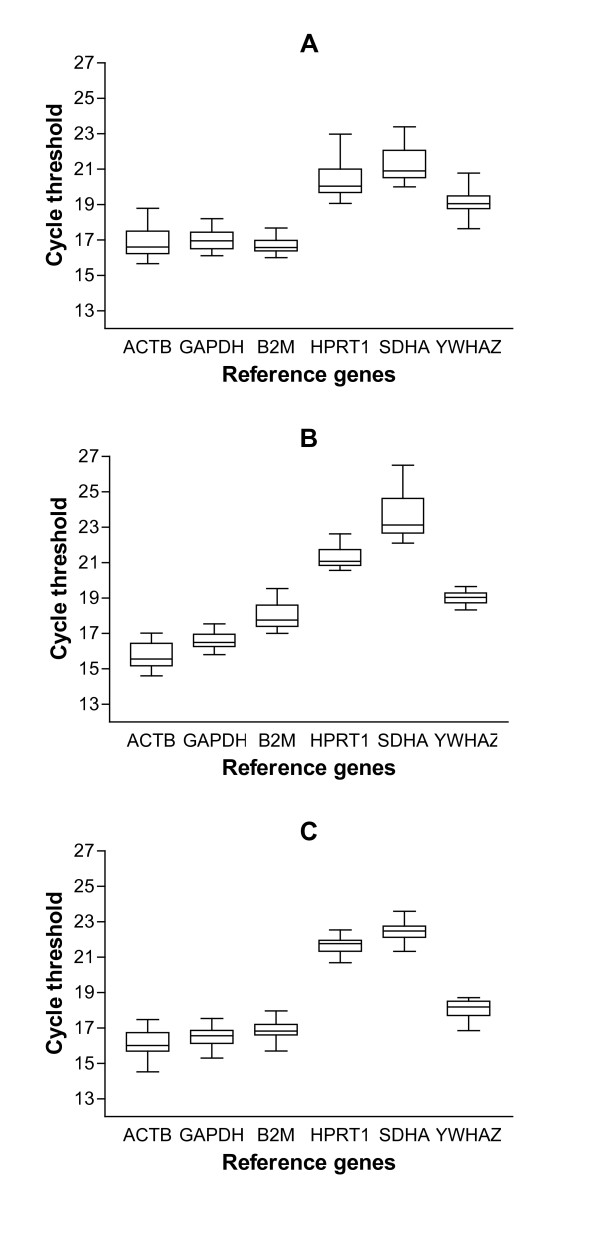
**qPCR cycle threshold values of 6 reference genes among 7 culture conditions (table 2)**. Distribution of expression levels of *ACTB, GAPDH, B2M, HPRT1, SDHA*, and *YWHAZ *in IL-1β treated C-28/I2 chondrocytes under the influence of glucosamine (A), curcumin (B) and diacerein (C) is shown as medians (lines), 25^th ^percentile to the 75^th ^percentile (boxes) and ranges (whiskers) (n = 63). Samples were normalized to the amount of total RNA.

A real-time qPCR assay, based on SYBR Green detection, was designed for the transcriptional profiling of six frequently used reference genes (*ACTB, GAPDH, B2M, HPRT1, SDHA, YWHAZ*) in the cDNA samples. Figures 1A, B and 1C show the distribution of the C_t _values of all samples under all culture conditions (Table 2) for glucosamine, curcumin and diacerein experiments, respectively. This 'whole error approach' is a simplified way to visualize the overall error introduced during the whole assay and gives an overview of the abundance of the genes in the samples. Since lower C_t _values indicate higher numbers of starting cDNA molecules, the most abundant reference genes in C-28/I2 cells were *ACTB*, *GAPDH *and *B2M *followed by *YWHAZ*. The amplification of *HPRT1 *and *SDHA *occurred about 5 C_t _values later. However, this 'whole error approach' does neither take into account the influence of qPCR efficiencies, nor does it provide satisfactory resolution for statistical evaluation of small differences between study groups. In a further step, the C_t _values of each sample were related to the average C_t _value of the respective untreated control group by the equation E^-ΔCt^. Tables 3, 4 and 5 show the relative reference gene expression levels of IL-1β treated chondrocytes under the influence of glucosamine, curcumin and diacerein, respectively. IL-1β did not provoke significant differences of any reference gene except for *ACTB *which was significantly up-regulated in the glucosamine (Table 3) and diacerein (Table 5) experiments (p < 0.05). Regarding the impact of the CPA used, a trend for dose-dependent down-regulation of all reference genes was observed for both the pre-treated and the simultaneously treated study groups. In contrast, time-dependent down-regulation seems to occur at the high concentrations of CPA used. We found that both glucosamine (Table 3) and curcumin (Table 4) treatment affected the expression level of all reference genes significantly (p < 0.05). Comparing two samples, a twofold change in gene expression level is commonly regarded as the benchmark of biological meaningfulness [29,35]. Therefore, a less than twofold change is considered as a requirement for suitability of a reference gene. In our study, a 48 h incubation of chondrocytes with high concentrations of glucosamine (Table 3) and curcumin (Table 4) (5 mM and 50 μM, respectively) induced most reference genes to exceed this benchmark of biological meaningfulness. The only exceptions were *B2M *in the case of glucosamine treatment (Table 3) and *YWHAZ *in the case of curcumin treatment (Table 4). The data further indicate differences in the reference gene regulation caused by different CPA. For diacerein-treated chondrocytes, *GAPDH, B2M, HPRT1 *and *SDHA *were not significantly affected by any culture condition used.

### GeNorm analysis

In order to determine the most stable reference genes in our study, the data were analyzed using the geNorm software. Table 6 shows the ranking of the 6 candidate reference genes according to their expression stability under the influence of glucosamine, curcumin and diacerein. The optimal number of reference genes necessary for the calculation of reliable normalization factors was assessed calculating the pairwise variations V_n_/V_n+1 _according to Vandesompele et al. [32]. RT-PCR normalization factors were calculated on the basis of the 3 most stable genes and stepwise inclusion of the other genes in the order of their expression stability. The pairwise variation V_n_/V_n+1 _was calculated between the 2 sequential normalization factors (NF_n _and NF_n+1_). A large variation means that the added gene has a significant effect on the newly calculated normalization factor and should preferably be included for calculation. In all 3 experiments, the inclusion of the 4^th ^reference gene did not contribute significantly to the variation of the normalization factor (V_3/4 _value < 0.15). Based on the proposed cut-off value of 0.15, below which the inclusion of an additional control gene is not required, the use of 3 control genes would therefore be sufficient for normalization in our study. The 3 most stable reference genes for glucosamine-treated samples were *GAPDH, B2M *and *SDHA*. In contrast, for curcumin and diacerein-treated samples, *HPRT1, GAPDH *and *B2M *provided the most stable expression levels with *SDHA *ranking the 6^th ^and 5^th ^positions, respectively. It is remarkable that *ACTB*, one of the most prominent and frequently used reference genes, proved to be one of the most variably expressed genes under the influence of selected CPA.

## Discussion

The claim of real-time qPCR to assess changes of mRNA expression levels in a quantitative way requires the choice of an adequate normalization strategy [26]. Normalization must be performed to compensate sample-to-sample and run-to-run variations. Reference genes theoretically represent a convenient way to normalize qPCR data since their use controls for all steps of gene expression analysis. However, the quality of normalized quantitative expression data cannot be better than the quality of the normalizer itself. Any variation in the normalizer will obscure real changes and produce additional errors. Therefore, the stability of the reference gene of choice represents the benchmark for the resolution of the qPCR assay and directly influences the significance of the results. By geometric averaging of multiple control genes, the geNorm software tool represents an alternative for accurate normalization of qPCR data, in particular, when the significance of subtle gene expression differences is evaluated [32]. The determination of the most stable reference genes in a given experimental setup and the subsequent calculation of normalization factors thus open up the possibility of reliably studying small expression differences even in low-copy number genes.

In the present study, we determined the expression stability of common reference genes under the influence of glucosamine, curcumin and diacerein in C-28/I2 chondrocytes. Immortalized C-28/I2 cells represent an alternative to primary human chondrocytes circumventing certain shortcomings of primary cells such as high inter-individual variability between different donors and dedifferentiation during in-vitro culture [39,40]. Moreover, C-28/I2 cells are known to be reactive to IL-1β [41] suggesting that they represent a suitable model for investigating the effects of CPA on the IL-1β-driven catabolic cascade. The present study design was developed to mirror experimental protocols that were used in recent studies [18-25] investigating the activity of CPA in chondrocytes in the presence of IL-1β. In order to create high variability, we used 3 different CPA for the treatment of chondrocyte cultures, i.e. glucosamine, curcumin and diacerein. The range of concentrations used in the presented experiments were in accordance with recently published in-vitro studies [20,23,42]. To mimic different approaches used previously, the CPA were added to the culture medium either simultaneously with IL-1β or prior to the addition of IL-1β.

The presented data report insignificant impact of IL-1β on the variability of most reference gene expression levels (p > 0.05). Only *ACTB *levels were significantly changed in the glucosamine and diacerein experiments. In this regard, the C-28/I2 model is in accordance with results of Chan et al. [25] who showed that *GAPDH *was not regulated by IL-1β in primary bovine chondrocytes. Moreover, our results demonstrated significantly variable expression levels of all reference genes in C-28/I2 chondrocytes under most culture conditions containing CPA. It is suggested that this variability was due to both dose and time dependent effects induced by the CPA. In an attempt to provide information on the most uniformly expressed reference genes for studies analyzing chondroprotective activity, we determined the ranking of 6 candidate genes according to their expression stability depending on the CPA under investigation. This ranking was not equivalent between the 3 different CPA. The high variability of *ACTB *in our study further points out the problem of relying on commonly used reference genes without an accurate validation process. Therefore, using the geNorm software, we defined the sets of reference genes suitable for studies on the presented CPA. *GAPDH, B2M *and *SDHA *are proposed for studies on glucosamine, while *HPRT1, GAPDH *and *B2M *can be used to investigate curcumin or diacerein activity. If glucosamine, curcumin and diacerein are to be compared, the same set as proposed for curcumin or diacerein can be used. It is generally recommended that the gene of interest and the selected reference gene should roughly be expressed at the same level. Therefore, the choice of a single reference gene (e.g. *GAPDH *or *SDHA *in glucosamine studies) may be determined by the abundance of the gene of interest. When a study aims at the fine measurement of small gene expression differences even the geometric mean of 2 or 3 reference genes could be of advantage.

## Conclusion

In conclusion, our report underlines the importance of accurate reference gene validation for each qPCR study. However, this validation process represents an evident disadvantage of the reference gene normalization strategy, since remarkable effort and costs are associated with its realization. In order to provide a reference for future studies on chondrocyte responses to different agents, we have defined the sets of suitable reference genes for the molecular biological investigation of glucosamine, curcumin and diacerein activities. Our findings will facilitate the use of the reference gene method for the normalization and data interpretation of CPA effects in the IL-1β stimulated chondrocyte model, in particular when subtle gene expression differences are to be investigated.

## Methods

### Materials

Glucosamine hydrochloride, curcumin, diacerein and 0.25 % Trypsin-EDTA were purchased from Sigma (St. Louis, MO, USA). Recombinant human IL-1β was obtained from Strathmann Biotec (Hamburg, D). Primers for real-time PCR experiments and the mi-Gel Extraction Kit were from Metabion (Martinsried, D). Dulbecco's modified Eagle's medium and 100x ITS solution were purchased from Gibco (Lofer, A). The StrataScript First-Strand Synthesis System and the Brilliant SYBR Green QPCR Master Mix were from Stratagene (La Jolla, CA, USA). All other chemicals were of analytical grade and obtained from Merck (Darmstadt, D) unless otherwise specified.

### Cell culture and experimental design

Immortalized C-28/I2 chondrocytes were used for all experiments in this study. In order to determine the influence of glucosamine, curcumin and diacerein on reference gene expression levels in chondrocytes, three sets of individual experiments – one for each compound – were performed.

2.3 × 10^5 ^chondrocytes were seeded into 25 cm^2 ^culture flasks (Corning, NY, USA) and cultured for 5 days in DMEM supplemented with 10 % fetal calf serum (Biochrom AG, Berlin, D), 2 μl/ml gentamycin and 50 μg/ml ascorbate. On days 5 and 6 the culture medium was changed to serum-free DMEM supplemented with 1 % (v/v) 100× ITS (10 μg/ml insulin + 5.5 μg/ml transferrin + 6.7 ng/ml sodium selenite) and 2 μl/ml gentamycin. Additionally, the particular CPA and/or IL-1β (10 ng/ml) were added as indicated in Table 2. Thereby, 7 different conditions for each CPA experiment were created: one control group without additional supplements, one IL-1β treated group, two groups simultaneously treated with both IL-1β and CPA at two different concentrations, two groups pre-incubated with CPA at two different concentrations prior to IL-1β addition, and one group treated with the respective CPA at the higher concentration without the addition of IL-1β. Each of these study groups consisted of three identically treated 'biological' replicates. Throughout cell culturing, cells were maintained in a humidified 5 % CO_2_/95 % air atmosphere at 37°C.

### RNA isolation and cDNA synthesis

On day 7 of the culture period, chondrocytes were harvested by trypsination using 0.05 % Trypsin-EDTA. The cells were washed with PBS and total RNA was extracted immediately using the NucleoSpin RNA II Kit (Macherey-Nagel, Dueren, D) according to the manufacturer's instructions. On-column DNase digestion was performed for all samples during the isolation process. To control for the integrity of the isolated RNA preparations, all samples were qualitatively assessed according to standard methods prior to use. Briefly, a 1.5% denaturing agarose gel was loaded with 2 μg of total RNA samples and stained with ethidium bromide after electrophoresis. The quantity of extracted RNA was determined using the Quant-It RiboGreen reagent (Molecular Probes, Eugene, USA) and the quantitative plate-read function of the Stratagene MxPro QPCR software. An R^2 ^> 0.990 of the standard curve plots was considered acceptable for accurate RNA quantification. Reverse transcription (RT) was performed using the StrataScript First-Strand Synthesis System, following the manufacturer's instructions. Briefly, 2 μg of total RNA were reverse transcribed in a 20 μl reaction volume using the manufacturer-supplied oligo(dT) primers. Minus RT controls were prepared for each sample using the identical procedure except for the omission of reverse transcriptase. All cDNA preparations and minus RT controls were diluted at a ratio of 1:10 with RNase free water prior to real-time PCR.

### Reference gene selection and primer optimization

Six reference genes (*ACTB, GAPDH, B2M, HPRT1, SDHA, YWHAZ*) belonging to different functional classes were selected to reduce the chance that these genes might be co-regulated (Table 1). Primers for *GAPDH *were designed using AlleleID 2.01 software (Premier Biosoft, CA, USA) and the Genbank sequence NM_002046. In order to avoid non-specific product formation, the primers were designed across exon junctions and with minimized self and cross dimer ΔG values. Additionally, BLAST analyses were performed to verify specificity. Primer sequences for all other reference genes were used as described by Vandesompele et al. [32]. Amplicon sizes were 140 bp for *ACTB*, 165 bp for *GAPDH*, 86 bp for *B2M*, 94 bp for *HPRT1*, 86 bp for *SDHA *and 94 bp for *YWHAZ*. Gene specific primers were used to amplify respective PCR products in order to provide sufficient template material for primer concentration optimization. PCR amplifications were performed using GoTaq DNA Polymerase (Promega, Wisconsin, USA) in a 50 μl reaction volume (10 μl of 5x Colourless Go Taq Reaction Buffer, 1 μl of PCR Nucleotide Mix, 5 μl of gene specific forward and reverse primer, 0.25 μl of GoTaq DNA polymerase, 2 μl of template DNA, and nuclease-free water to 50 μl). The PCR program consisted of an initial denaturation step at 95°C for 2 min, followed by 35 cycles at 95°C for 45 s, at 55°C for 45 s and at 72°C for 1 min, and a final extension step at 72°C for 5 min. Amplified PCR products were diluted with bromophenol blue solution at a ratio of 1:4 and purified by gel electrophoresis using a 1% agarose gel. After staining with ethidium bromide, the designated bands were cut out of the agarose gel and the products were extracted from the gel using the mi-Gel Extraction Kit according to the manufacturer's instructions. Optimization of primer concentrations for the qPCR experiments was performed by qPCR assays as described below using different primer concentrations and a 1:10^3 ^dilution of purified PCR products as templates. Optimal primer pairs for each reference gene were identified selecting the combination that gave the lowest C_t _value and the highest ΔR_n _value.

### Real-time qPCR

All qPCR reactions were performed in 25 μl reaction mixtures containing 1 μl cDNA, 12.5 μl Brilliant SYBR Green QPCR Master Mix, primer pairs as listed in Table 1, and nuclease-free water to 25 μl. Each biological replicate was run in triplicate on a Mx3000P QPCR system (Stratagene). Thermocycling conditions consisted of an initial polymerase activation step at 95°C for 10 min, followed by 35 cycles at 95°C for 30 s, at 55°C for 1 min, and at 72°C for 1 min. Afterwards, melting curves were generated to confirm a single gene-specific peak and to detect primer-dimer formation by heating the samples stepwise from 55°C to 95°C while continuously monitoring the fluorescence. NTC were included in each run to control for contaminations. Minus RT controls were run to verify the efficiency of the on-column DNase digestion. The results were analyzed using the MxPro real-time QPCR software (Stratagene). Baselines and thresholds were automatically set by the software and used after manual inspection. The crossing point of the amplification curve with the threshold represented the cycle threshold (C_t_). PCR efficiencies for each primer pair were derived from standard curves using five-fold dilution series covering a 4 to 5 log dynamic range starting from one randomly selected undiluted cDNA sample of the untreated control group. The reactions were run in duplicate and no-template controls were included.

### Data presentation and calculations

Results were exported to Microsoft Excel and GraphPad Prism for further analyses. For each test compound, the distribution of the expression levels (C_t _values) for each reference gene under the 7 experimental conditions was displayed as Box and Whiskers plots. In a next step, data within each experiment were normalized to the average level of mRNA expression (C_t _value) of the respective control group using the relationship E^-ΔCt ^(E, efficiency; ΔC_t_, difference between C_t _values). Statistics were performed using one-way ANOVA with post-hoc Dunnett tests comparing each study group with the untreated control group (significance p < 0.05). The geNorm application software for Microsoft Excel was additionally used as described by Vandesompele et al. [32] to identify the most stable reference gene under the described conditions, and to determine the optimal number of reference genes required for reliable normalization of qPCR data.

## Authors' contributions

ST conceived of the study, participated in the qPCR experiments, carried out the statistical analyses and drafted the manuscript. WH and CP carried out the cell culture and gene expression analyses. MBG contributed to writing the manuscript. MW, FMU, FG and HV supervised the study design and the experimental processes. All authors read and approved the manuscript.

## Figures and Tables

**Table 1:** Function of reference genes, primer sequences, optimal primer concentrations and reaction efficiencies of qPCR experiments.

**Symbol**	**Function**	**Primer sequence**	**nM**	**Efficiency**
ACTB	Cytoskeletal structural protein	fw: CTGGAACGGTGAAGGTGACA rv: AAGGGACTTCCTGTAACAATGCA	300 100	1.94 ± 0.01
GAPDH	Oxidoreductase in glycolysis and gluconeogenesis	fw: GGAGTCCACTGGCGTCTTCAC rv: GAGGCATTGCTGATGATCTTGAGG	300 600	1.94 ± 0.01
B2M	Beta-chain of major histocompatibility complex class I molecules	fw: TGCTGTCTCCATGTTTGATGTATCT rv: TCTCTGCTCCCCACCTCTAAGT	300 900	2.03 ± 0.04
HPRT1	Purine synthesis in salvage pathway	fw: TGACACTGGCAAAACAATGCA rv: GGTCCTTTTCACCAGCAAGCT	300 900	1.94 ± 0.03
SDHA	Electron transporter in the TCA cycle and respiratory chain	fw: TGGGAACAAGAGGGCATCTG rv: CCACCACTGCATCAAATTCATG	300 900	2.00 ± 0.01
YWHAZ	Signal transduction by binding to phosphorylate serine residues	fw: ACTTTTGGTACATTGTGGCTTCAA rv: CCGCCAGGACAAACCAGTAT	600 600	1.98 ± 0.01

Optimal primer concentrations were determined using a matrix of forward and reverse primers varying in concentration from 50 nM to 900 nM. Combinations that gave the lowest C_t _value and the highest ΔR_n _value were selected. qPCR efficiencies for each primer pair were derived from standard curves (n = 3) using five-fold dilution series covering a 4 to 5 log dynamic range starting from one randomly selected undiluted cDNA sample of the untreated control group.

**Table 2:** Experimental design

Experimental condition	Supplements on days of culture	
	
	day 5	day 6
Control	-	-
IL-1β	-	IL-1β
Simultaneous incubation with CPA [c]_1_	-	[c]_1_+IL-1β
Simultaneous incubation with CPA [c]_2_	-	[c]_2_+IL-1β
Pre-incubation with CPA [c]_1_	[c]_1_	[c]_1_+IL-1β
Pre-incubation with CPA [c]_2_	[c]_2_	[c]_2_+IL-1β
CPA only	[c]_2_	[c]_2_

Supplements added to the chondrocyte culture medium on days 5 and 6 of the culture period are presented. Either glucosamine, curcumin or diacerein were used as chondroprotective agent (CPA) in 3 separate experiments. Each experiment followed the presented setup of 7 different study groups.

IL-1β, 10 ng/ml recombinant human interleukin-1beta; [c]_1_, low concentration of glucosamine (50 μM), curcumin (5 μM) or diacerein (5 μM); [c]_2_, high concentration of glucosamine (5 mM), curcumin (50 μM) or diacerein (50 μM).

**Table 3:** Relative mRNA levels of C-28/I2 chondrocytes under the influence of IL-1β and glucosamine.

**Experimental condition**	**mRNA relative to untreated control group (mean ± SD)**					
	
	**ACTB**	**GAPDH**	**B2M**	**HPRT1**	**SDHA**	**YWHAZ**
IL-1β	**1.30 ± 0.30**	1.05 ± 0.27	1.14 ± 0.22	1.08 ± 0.24	1.00 ± 0.20	0.88 ± 0.16
Simultaneous incubation with glucosamine [c]_1_	1.27 ± 0.24	1.13 ± 0.18	**1.35 ± 0.22**	0.93 ± 0.13	0.83 ± 0.14	0.83 ± 0.15
Simultaneous incubation with glucosamine [c]_2_	**0.63 ± 0.16**	**0.69 ± 0.09**	1.04 ± 0.11	**0.56 ± 0.06**	**0.77 ± 0.15**	**0.61 ± 0.11**
Pre-incubation with glucosamine [c]_1_	0.87 ± 0.11	0.97 ± 0.25	1.11 ± 0.22	**0.66 ± 0.16**	1.02 ± 0.11	0.74 ± 0.10
Pre-incubation with glucosamine [c]_2_	0.28 ± 0.05*	0.43 ± 0.07*	**0.67 ± 0.15**	0.37 ± 0.08*	0.35 ± 0.06*	0.37 ± 0.11*
Glucosamine only	**0.60 ± 0.27**	**0.66 ± 0.22**	0.77 ± 0.34	0.15 ± 0.05*	0.20 ± 0.07*	0.37 ± 0.28*

Boldfaced values p < 0.05

*p < 0.05 and change > 2-fold

mRNA levels under the treatment with glucosamine in different experimental conditions are presented relative to the untreated control group calculated by the equation E^-ΔCt ^(Mean ± SD, n = 9; value of 1 represents average level of mRNA expression in untreated control group). [c]_1_, low concentration of glucosamine (50 μM); [c]_2_, high concentration of glucosamine (5 mM).

**Table 4:** Relative mRNA levels of C-28/I2 chondrocytes under the influence of IL-1β and curcumin.

**Experimental condition**	**mRNA relative to untreated control group (mean ± SD)**					
	
	**ACTB**	**GAPDH**	**B2M**	**HPRT1**	**SDHA**	**YWHAZ**
IL-1β	0.93 ± 0.16	0.86 ± 0.10	1.06 ± 0.17	0.97 ± 0.15	0.91 ± 0.11	0.92 ± 0.13
Simultaneous incubation with curcumin [c]_1_	**0.72 ± 0.07**	**0.74 ± 0.11**	**0.85 ± 0.10**	0.88 ± 0.11	**0.76 ± 0.10**	**0.78 ± 0.08**
Simultaneous incubation with curcumin [c]_2_	0.41 ± 0.04*	0.89 ± 0.16	**0.64 ± 0.06**	**0.86 ± 0.08**	**0.61 ± 0.06**	**0.85 ± 0.11**
Pre-incubation with curcumin [c]_1_	0.87 ± 0.13	0.98 ± 0.15	0.94 ± 0.18	0.97 ± 0.11	0.95 ± 0.23	1.02 ± 0.21
Pre-incubation with curcumin [c]_2_	0.37 ± 0.06*	0.46 ± 0.05*	0.28 ± 0.04*	0.38 ± 0.04*	0.09 ± 0.02*	**0.65 ± 0.06**
Curcumin only	0.28 ± 0.01*	0.48 ± 0.06*	0.31 ± 0.03*	0.38 ± 0.05*	0.10 ± 0.01*	**0.63 ± 0.04**

Boldfaced values p < 0.05

* p < 0.05 and change > 2-fold

mRNA levels under the treatment with curcumin in different experimental conditions are presented relative to the untreated control group calculated by the equation E^-ΔCt ^(Mean ± SD, n = 9; value of 1 represents average level of mRNA expression in untreated control group). [c]_1_, low concentration of curcumin (5 μM); [c]_2_, high concentration of curcumin (50 μM).

**Table 5:** Relative mRNA levels of C-28/I2 chondrocytes under the influence of IL-1β and diacerein.

**Experimental condition**	**mRNA relative to untreated control group (mean ± SD)**					
	
	**ACTB**	**GAPDH**	**B2M**	**HPRT1**	**SDHA**	**YWHAZ**
IL-1β	**1.5 ± 0.63**	1.2 ± 0.57	1.10 ± 0.08	1.23 ± 0.46	1.02 ± 0.66	0.89 ± 0.45
Simultaneous incubation with diacerein [c]_1_	1.03 ± 0.39	1.11 ± 0.40	1.36 ± 0.29	1.26 ± 0.40	1.13 ± 0.34	1.14 ± 0.43
Simultaneous incubation with diacerein [c]_2_	0.71 ± 0.18	1.02 ± 0.21	1.15 ± 0.20	1.07 ± 0.25	0.88 ± 0.17	0.69 ± 0.17
Pre-incubation with diacerein [c]_1_	1.21 ± 0.21	1.12 ± 0.33	1.23 ± 0.57	1.29 ± 0.40	1.35 ± 0.39	1.11 ± 0.31
Pre-incubation with diacerein [c]_2_	**0.56 ± 0.07**	0.79 ± 0.11	0.96 ± 0.12	1.00 ± 0.13	0.98 ± 0.19	0.7 ± 0.15
Diacerein only	0.43 ± 0.06*	0.63 ± 0.10	0.73 ± 0.09	0.87 ± 0.15	0.88 ± 0.15	**0.59 ± 0.07**

Boldfaced values p < 0.05

* p < 0.05 and change > 2-fold

mRNA levels under the treatment with diacerein in different experimental conditions are presented relative to the untreated control group calculated by the equation E^-ΔCt ^(Mean ± SD, n = 9; value of 1 represents average level of mRNA expression in untreated control group). [c]_1_, low concentration of diacerein (5 μM); [c]_2_, high concentration of diacerein (50 μM).

**Table 6:** Ranking of the reference genes as determined using the geNorm software tool.

Glucosamine	Curcumin	Diacerein
**GAPDH/B2M**	**HPRT1/GAPDH**	**HPRT1/GAPDH**
SDHA	**B2M**	**B2M**
**HPRT1**	ACTB	YWHAZ
ACTB	YWHAZ	SDHA
YWHAZ	SDHA	ACTB

The genes are ranked in order of their expression stability under the influence of glucosamine, curcumin and diacerein decreasing from top to bottom. The 2 most stable reference genes in each experiment cannot be ranked in order due to the required use of ratios for the calculation of gene expression stability. The reference genes proposed for comparing the 3 chondroprotective agents are displayed in bold letters.
